# The role of self-compassion in the relationship between pain and depression in palliative patients

**DOI:** 10.1192/j.eurpsy.2021.1148

**Published:** 2021-08-13

**Authors:** J. Guerra, L. Santiago, D. Carreiras

**Affiliations:** 1 Faculdade De Medicina Da Universidade De Coimbra, Universidade de Coimbra, Coimbra, Portugal; 2 Center For Research In Neuropsychology And Cognitive And Behavioral Intervention, University of Coimbra, Coimbra, Portugal

**Keywords:** Pain, Depression, Palliative patients, Self-compassion

## Abstract

**Introduction:**

In palliative care, depression and pain are prevalent variables with a reciprocal and controversial relationship. Depression is common in people with chronical diseases. In the last decades, self-compassion has been pointed as a protective psychological process to negative affect.

**Objectives:**

The current study aimed to test the role of self-compassion in the relationship between pain and depression in palliative patients.

**Methods:**

Sample was composed of 33 patients in palliative care, with a mean of 74.12 years of age (SD = 12.76). Participants completed self-report questionnaires and data was analyzed using SPSS.

**Results:**

From the descriptive analysis of the results of the Geriatric Depression Scale, 22 patients were depressed (66.6%), 9 of them in severe depression (27.7%). Self-compassion presented a negative and moderate correlation with depression. Depression was positively correlated with pain. A hierarchical regression to predict depression was conducted. Firstly, pain was entered as a predictive variable with a significant effect. Secondly, self-compassion was entered, and the model was significantly incremented, explaining 41% of depression. Only self-compassion was significant in this model.

**Conclusions:**

Discussion and conclusion: The association between pain and depression in palliative care corroborate previous research. Results seemed to show that self-compassion has a significant effect in the relationship between pain and depression. Cultivating a compassion self-to-self relationship might have an important effect attenuating the link between pain and depression in palliative care.

